# Role of Proteolipid Protein in HSV-1 Entry in Oligodendrocytic Cells

**DOI:** 10.1371/journal.pone.0147885

**Published:** 2016-01-25

**Authors:** Raquel Bello-Morales, Antonio Jesús Crespillo, Beatriz Praena, Enrique Tabarés, Yolanda Revilla, Elena García, Alberto Fraile-Ramos, Wia Baron, Claude Krummenacher, José Antonio López-Guerrero

**Affiliations:** 1 Universidad Autónoma de Madrid, Departamento de Biología Molecular, Edificio de Biología, Darwin 2, Cantoblanco, 28049, Madrid, Spain; 2 Universidad Autónoma de Madrid, Facultad de Medicina, Arzobispo Morcillo 4, 28029, Madrid, Spain; 3 Centro de Biología Molecular Severo Ochoa, CSIC-UAM, Nicolás Cabrera 5, Cantoblanco, 28049, Madrid, Spain; 4 Universidad Complutense de Madrid, Facultad de Medicina, Plaza de Ramón y Cajal, s/n Ciudad Universitaria, 28040, Madrid, Spain; 5 University of Groningen, Faculty of Medical Sciences, Antonius Deusinglaan 1, 9713 AV, Groningen, The Netherlands; 6 Department of Biological Sciences and Department of Biomedical and Translational Sciences, Rowan University, Glassboro, NJ, 08028, United States of America; University of Illinois at Chicago, UNITED STATES

## Abstract

Herpes simplex virus type 1 (HSV-1) has the ability to enter many different hosts and cell types by several strategies. This highly prevalent alphaherpesvirus can enter target cells using different receptors and different pathways: fusion at a neutral pH, low-pH-dependent and low-pH-independent endocytosis. Several cell receptors for viral entry have been described, but several observations suggest that more receptors for HSV-1 might exist. In this work, we propose a novel role for the proteolipid protein (PLP) in HSV-1 entry into the human oligodendrocytic cell line HOG. Cells transfected with PLP-EGFP showed an increase in susceptibility to HSV-1. Furthermore, the infection of HOG and HOG-PLP transfected cells with the R120vGF virus–unable to replicate in ICP4-defficient cells- showed an increase in viral signal in HOG-PLP, suggesting a PLP involvement in viral entry. In addition, a mouse monoclonal antibody against PLP drastically inhibited HSV-1 entry into HOG cells. PLP and virions colocalized in confocal immunofluorescence images, and in electron microscopy images, which suggest that PLP acts at the site of entry into HOG cells. Taken together these results suggest that PLP may be involved in HSV-1 entry in human oligodendrocytic cells.

## Introduction

Herpes simplex virus type 1 (HSV-1) is a highly prevalent human pathogen belonging to the neurotropic alphaherpesviruses. HSV-1 infects epithelial cells and establishes latency in neurons in sensory ganglia [1, 2], but is also capable of spreading to the central nervous system (CNS) and causing meningitis or encephalitis [3].

Heparan sulfate glycosaminoglycans act as attachment receptors for the viral glycoprotein gC [4]. Although gC is not essential for viral entry, its absence decreases infectivity, due to a reduced efficiency of viral binding to cells [5]. In the absence of gC, gB can mediate attachment to heparan sulfate [3]. Four viral glycoproteins, gB, gD, gH, and gL are necessary for viral entry into cells [5, 6]. HSV gD binding to its receptors triggers the viral membrane fusion process which requires the heterodimer gH/gL and the fusion protein gB. Fusion of the viral envelope may occur with the plasma membrane in a pH-independent manner or with endosomal membrane after endocytosis [7, 8] to deliver the nucleocapsid and tegument into the cell cytoplasm. The major entry receptors for gD include HVEM [9], nectin-1 [10] and 3-O-sulfated heparan sulfate [11]. HVEM (herpesvirus entry mediator) is a member of the TNF receptor family expressed in several tissues and cell types, including epithelial cells, fibroblasts, monocytes and lymphocytes [9, 12, 13]. Nectins are molecules that mediate cell-cell adhesion in adherens junctions [14]. They are also expressed in a variety cell types, including epithelial cells, fibroblasts and neurons [15, 16]. A third viral receptor, 3-O-sulfated heparan sulfate, which appears when certain D-glucosaminyl-3-O-sulfotransferases modify heparan sulfate, has been shown to be active in CHO cells [11]. Other HSV-1 gB receptors, which have been found out recently, include paired immunoglobulin-like type 2 receptor (PILR) alpha [17] and myelin-associated glycoprotein (MAG) [18]. It has been recently reported that the interaction of HSV gH/gL heterodimer with its receptor αvβ6- or αvβ8-integrin results in the dissociation of gL from the heterodimer and its release in the medium, a process that requires the presence of gD, nectin1, and gB [19].

The broad range of animal species infectable by HSV-1 suggests that surface receptors for this virus are highly conserved or that different receptors might be used by HSV to enter different cell types [9, 20]. Indeed, data show that utilization of alternative receptors by HSV-1 is quite significant, since it can use different receptors according to the target cell [12]. Moreover, HSV-1 can also enter different cell types not only using different receptor, but also by different pathways: in many cultured cell lines, such as Vero and HEp-2, HSV-1 enters cells by a pH-neutral fusion with the cell surface, but entry into HeLa and CHO-K1 cells does depend on endocytosis and subsequent exposure to a low pH [8].

Oligodendrocytes (OLs) are the glial cells that produce myelin,–the electrically insulating layer that surrounds axons [21, 22]–in the CNS [23]. Proteolipid protein (PLP), together with DM20, a smaller isoform generated by alternative splicing, are the most abundant proteins in the CNS myelin, comprising around the 50% of total myelin proteins [24]. PLP has a crucial structural role in maintaining the stability of the intraperiod lines of compact myelin [25, 26] although other nonstructural roles for this protein have been also proposed [27, 28].

Previous work carried out by our laboratory has shown that both nectin-1 and HVEM are functioning as HSV-1 receptors in HOG cells and that the virus can follow an endocytic pathway to infect these cells. Moreover, we have also reported an increase in PLP levels in cells infected with K26GFP [29]. In the present work, we propose that PLP plays a role in HSV-1 entry into the human HOG oligodendrocytic cell line. Several pieces of evidence support this novel role for PLP. First, cells transfected with PLP-EGFP showed an increase in susceptibility to HSV-1. Second, antibodies directed to PLP significantly blocked viral entry. Third, the increased infection of HOG-PLP cells compared to HOG cells by a replication-deficient ICP4-negative virus (R120vGF) confirms that PLP plays a role early in infection. Finally, PLP colocalized with virions in electron microscopy images and by immunofluorescence (when HSV glycoprotein gD is stained). Taken together these results suggest that PLP could participate in a new way of viral entry in oligodendrocityc cells.

## Materials and Methods

### Antibodies, reagents and plasmids

Horseradish peroxidase-conjugated secondary anti-IgG antibodies were from Millipore (Billerica, MA, USA). Anti-GFP rabbit polyclonal serum A6455, Alexa 488-, Alexa 647- and Alexa 594-conjugated secondary antibodies were obtained from Molecular Probes (Eugene, OR, USA). Low-glucose DMEM, fetal bovine serum (FBS), o-nitrophenyl-β-D-galactopyranoside (ONPG), carboxymethylcellulose sodium salt (CMC) medium-viscosity and protease inhibitor cocktail were purchased from Sigma Chemical Co. (St. Louis, MO, USA). Mowiol was from Calbiochem (Merck Chemicals, Germany). Jet-PEI was from Polyplus-transfection (Illkirch, France).

PLP-EGFP was generated by fusing EGFP to the COOH terminus of PLP by gene fusion PCR. The fusion product was cloned into pEGFPN1 vector using the EcoRI–NotI site. Stable cell lines were obtained by the cotransfection of PLP-EGFPNI and pMSCV-hygro (CLONTECH Laboratories, Inc.) followed by the selection of clones by incubation with hygromycin.

### Cell lines and virus

The human HOG cell line, established from a surgically removed human oligodendroglioma [30] was kindly provided by Dr. A. T. Campagnoni (University of California, UCLA, USA). Cells were cultured on Petri dishes in GM containing low-glucose DMEM supplemented with 10% heat-inactivated fetal bovine serum (FBS), penicillin (50 U/mL) and streptomycin (50 μg/mL) at 37°C in an atmosphere of 5% CO_2_. Differentiation medium (DM) consisted of serum-free low-glucose DMEM supplemented with additives [31].

To produce a PLP-EGFP stably-expressing cell line HOG cells were stably transfected with PLP-EGFP plasmid. Twenty-four hours prior transfection of the HOG cell line, 10^6^ cells were plated in 100 mm Petri dishes with GM. Cells were transfected with 8 μg of DNA, using the JetPEI reagent according to the manufacturer’s instructions. Cells were incubated with DNA for 24 h in GM and, 48 h after transfection, selection of stable HOG cell transfectants was initiated by treatment with 1 mg/ml G418 sulfate.

R120vGF is an EGFP-expressing recombinant virus containing *vhs* gene and inmediate early genes except ICP4 [29]. This virus was propagated in E5 cells, a Vero cell line expressing the ICP4 protein of HSV-1 [32]. After entry into cells, R120vGF virus expresses EGFP and immediate early proteins, but it is not able to complete the viral cycle due to the abscence of ICP4. K26GFP virus was a kind gift of Dr. Desai (Johns Hopkins University, Baltimore, USA). It was obtained by fusing GFP to the HSV-1 capsid protein VP26 [33]. K26GFP and wild type HSV-1 (F strain) viruses were propagated and titrated on Vero cells. CHO-K1 cells were a kind gift of Dr. R. Longnecker (Northwestern University, Chicago, USA).

### Viral infections

For viral infection assays, 2x10^6^ HOG cells growing in 25 cm^2^ flasks were mock infected or infected with the corresponding virus. During viral adsorption, cells were maintained in DMEM with antibiotics in the absence of FCS. Subsequently, cultures were rinsed and cultured in its corresponding medium. Viral titer was quantified by an endpoint dilution assay determining the 50% tissue culture infective dose (TCID_50_) in Vero cells, considering the final dilution that shows cytopathic effect and using the Reed and Muench method.

For plaque assay, confluent monolayers of cells plated in 6-well tissue culture dishes were infected with serial dilutions of HSV-1. After viral adsorption, cells were washed and overlayed with CMC. The CMC solution was prepared in distilled water at 2% (w/v) and stirred at room temperature for one hour. CMC overlay (1% final concentration) was prepared by mixing equal volumes of CMC 2% and medium double-strength. Two millilitres of CMC overlay were added to each well. Plates were incubated at 37°C in a humidified 5% CO_2_ incubator for 48 hours. After that, CMC overlay was aspirated and cells were washed with PBS. Plaques were visualized by staining with crystal violet.

### Viral entry assay

To determine HSV-1 entry, confluent monolayers of HOG cells plated in 96-well tissue culture dishes were infected with a recombinant HSV-1 (KOS) gL86, which expresses β-galactosidase upon entry into cells. After 6 h p.i., β-galactosidase assays were performed using a soluble substrate ONPG assay. The enzymatic activity was measured at 410 nm using a Benchmark microplate reader (Bio Rad). HSV-1 resistant CHO-K1 cells were used as control.

### Antibody blocking assay

HOG cells cultured in DM and plated in 24-well plates were preincubated at room temperature with twofold dilutions of a mouse monoclonal anti-PLP antibody for 90 min. Cells were then challenged with identical doses of K26GFP at 5 × 10^5^ pfu per well at 37°C. After 20 hours, the cells were washed twice with PBS and treated for flow cytometry. Mouse serum was used as a control.

### Immunoblot analysis

Samples were subjected to SDS-PAGE in 10% acrylamide gels under reducing conditions and transferred to Immobilon-P membranes (Millipore). After blocking with 5% nonfat dry milk, 0.05% Tween 20 in PBS, blots were incubated for 1 h at room temperature with primary antibodies. After several washes with 0.05% Tween 20 in PBS, blots were incubated for 1 h with secondary antibodies coupled to horseradish peroxidase, washed extensively, and developed using an enhanced chemiluminescence Western blotting kit (ECL, Amersham, UK).

### Flow Cytometry Analysis

To perform FACS analysis, cells were dissociated by incubation for 1 minute in 0.05% trypsin/0.1% EDTA (Invitrogen) at room temperature and washed and fixed in 4% paraformaldehyde for 15 minutes. Then, cells were rinsed and resuspended in PBS. Cells were analyzed using a FACSCalibur Flow Cytometer (BD Biosciences).

### Immunofluorescence microscopy

Cells cultured on DM and grown on glass coverslips were fixed in 4% paraformaldehyde for 20 min and rinsed with PBS. Cells were then permeabilized with 0.2% Triton X-100, rinsed and incubated for 30 min with 3% bovine serum albumin in PBS with 10% human serum, to block the HSV-1-induced IgG Fc receptors. For double and triple-labeled immunofluorescence analysis, cells were incubated for 1 h at room temperature with the appropriate primary antibodies, rinsed several times and incubated at room temperature for 30 min with the relevant fluorescent secondary antibodies. Antibodies were incubated in the presence of 10% human serum. Controls to assess labeling specificity included incubations with control primary antibodies or omission of the primary antibodies. After thorough washing, coverslips were mounted in Mowiol. Images were obtained using an LSM510 META system (Carl Zeiss) coupled to an inverted Axiovert 200 microscope. Processing of confocal images was made by FIJI-ImageJ software.

### Immunogold-labelling EM

HOG-PLP cells were mock-infected or infected with HSV-1 at a m.o.i. of 100 and incubated for 1 h at 4°C. Then cells were washed with PBS and fixed in 4% paraformaldehyde in 0.1 M sodium phosphate buffer, pH 7.4, at 4°C for 2 hour. After that, cells were incubated overnight with 8% paraformaldehyde in 0.1 M sodium phosphate buffer, pH 7.4, at 4°C. Fixed cells were washed in PBS containing 20 mM glycine and embedded in 12% gelatine, infiltrated with 2.3 M sucrose and frozen in liquid nitrogen. Cryosections were stained with a mouse monoclonal anti-PLP antibody. Primary antibody was detected with 15 nm anti-mouse-gold (British BioCell, Cardiff, UK). Cryosections were examined with a JEM1010 transmission EM (Jeol, Tokyo, Japan).

## Results

### PLP overexpression increases HSV-1 infection in HOG cells

**Fig 1:**
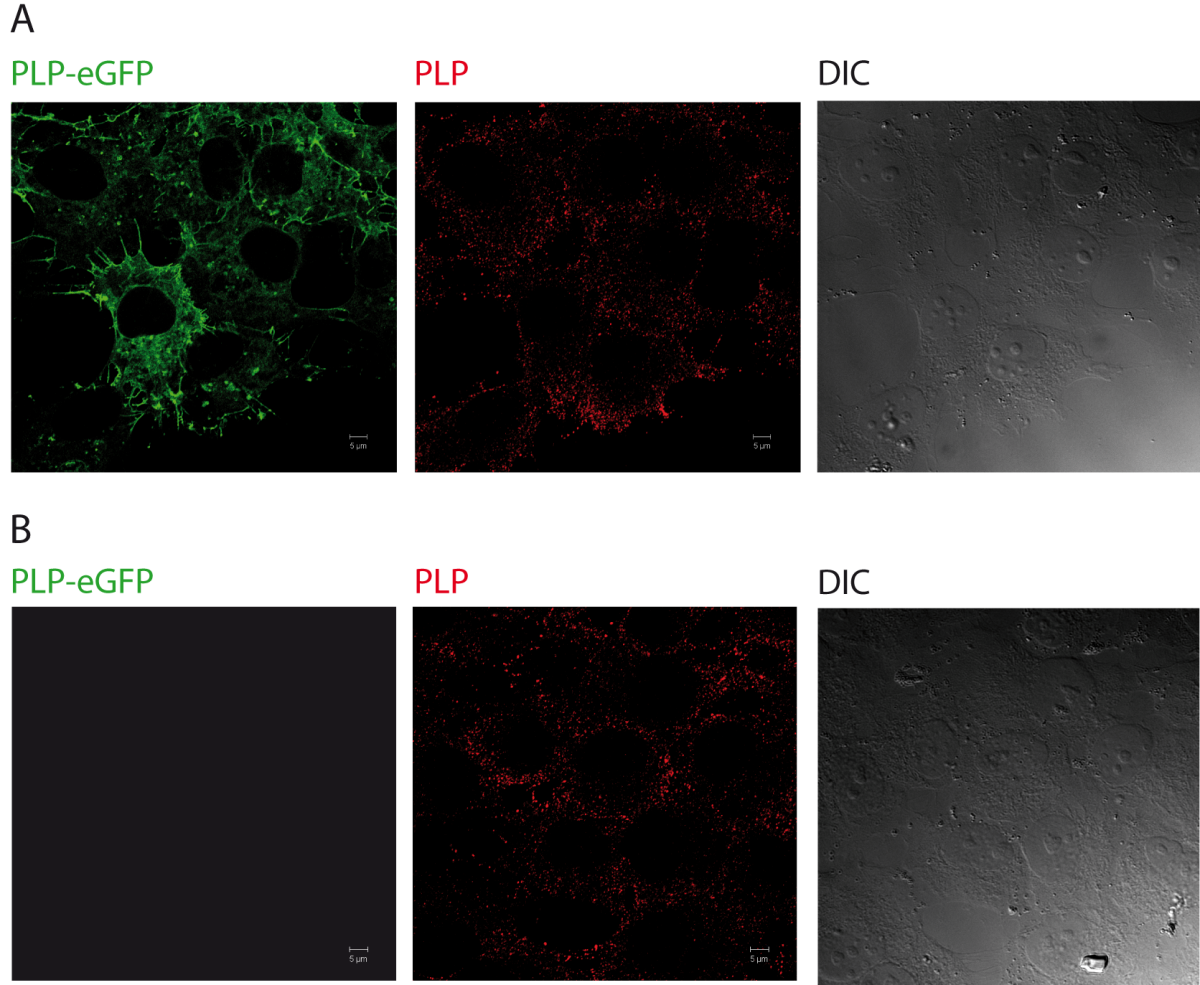
Expression of PLP-EGFP in HOG cells. Cells stably transfected (A) or mock-transfected (B) with PLP-EGFP were fixed and processed for confocal indirect immunofluorescence analysis with a polyclonal anti-PLP antibody. As the images show, the localization pattern of exogenous PLP (green) is similar to that of endogenous PLP (red). (DIC: Differential Interference Contrast).

**Fig 2:**
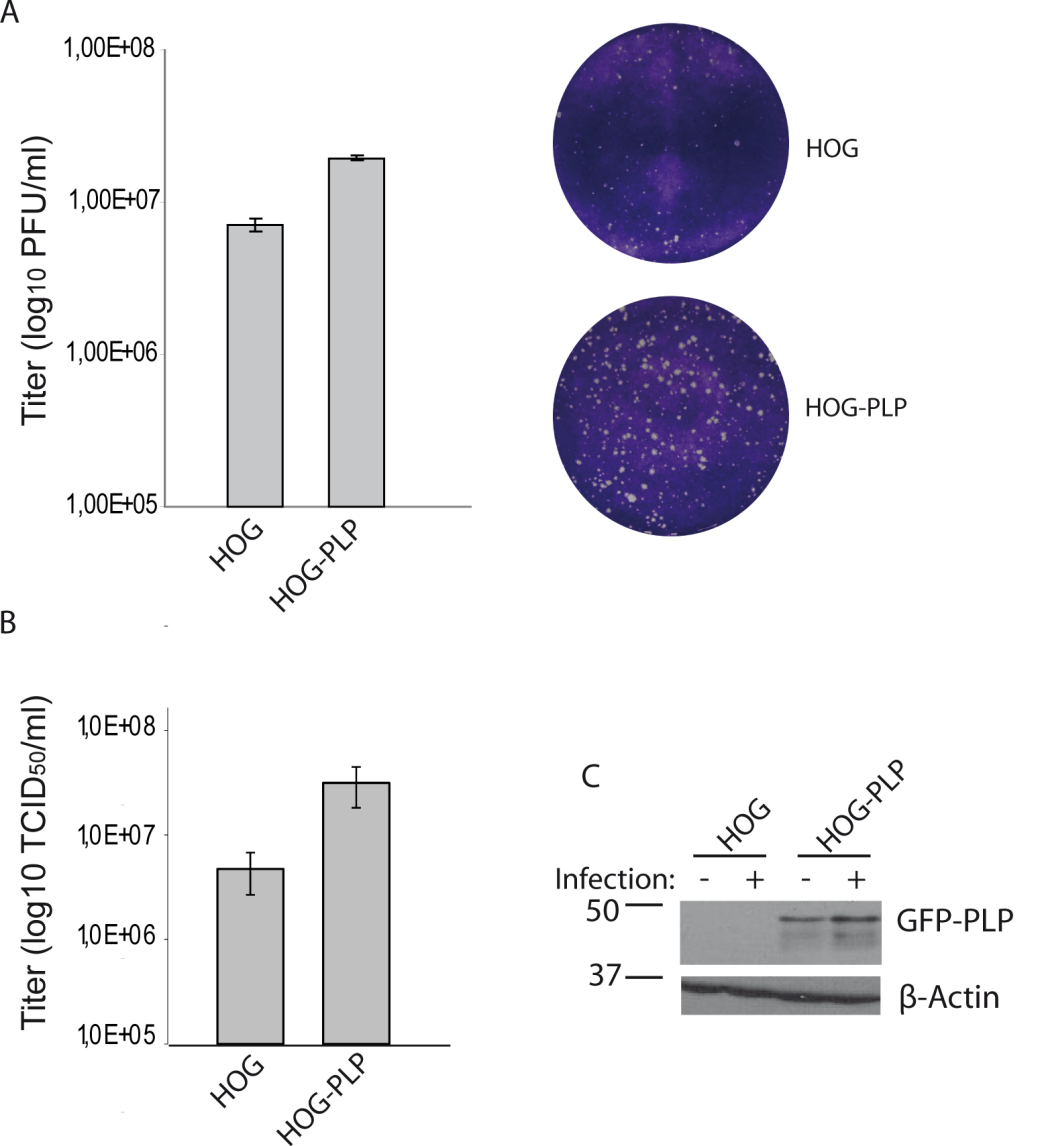
Effect of PLP overexpression on HSV-1 infection. HOG and HOG-PLP cells were infected with HSV-1. PLP-transfected HOG cells showed higher susceptibility to HSV-1 than mock-transfected cells (A and B). A. Plaque assay showed an increase in the number of plaque forming units (p.f.u.) per ml in PLP-transfected cells compared to mock-transfected control cells. Two representative wells are also shown. The average plaque size of infected HOG-PLP cells is slightly larger than that of plaques in HOG cells. B. Cells were infected at a m.o.i. of 0.1 with HSV-1, and viral titers were determined 20 h p.i. by TCID_50_. Virus yield was significantly increased in PLP-transfected cells. The increment on viral yield correlated with the presence of PLP-EGFP, as shown by immunoblotting (C) with an anti-GFP antibody. C. HOG and HOG-PLP cells were infected with HSV-1 at an m.o.i. of 0,1. After 24 h p.i., equal number of cells were subjected to SDS–PAGE and analyzed by immunoblotting with a rabbit polyclonal anti-GFP antibody.

The role of PLP in the infection of the human oligodendrocytic HOG cells was tackled in this study. First of all, to assess whether overexpression of PLP was correlated with an increase in HSV-1 infection, we produced a PLP-EGFP-expressing HOG cell line named HOG-PLP (Fig 1). To address the role of PLP in HSV plaque formation, confluent monolayers of HOG cells were infected with serial dilutions of HSV-1 as previously described [29]. A significantly (p<0.01) larger number of plaques was counted in HOG-PLP (a 2,7 times increment) compared to HOG cells when cells were infected with the same viral dose (Fig 2A). Furthermore, the average plaque size of infected HOG-PLP cells was slightly larger than that of plaques in HOG cells, suggesting that PLP could also be affecting viral spread. Additionally, HOG and HOG-PLP cells were infected at a m.o.i of 1 with HSV-1 and the viral progeny was titrated by determining the TCID_50_/ml. Viral yield of infected HOG-PLP cells was significantly (p<0.01) higher compared to that of HOG non-transfected cells (Fig 2B). PLP-EGFP overexpression led to a 6 fold increase in viral yield. This increase in viral progeny production correlated with the presence of PLP-EGFP (Fig 2C). In addition, Fig 2C shows an increase in PLP-EGFP in HSV-1 infected cells, which coincides with our previous results. GFP-MAL2/HOG cell line overexpressing exogenous MAL2 protein [34], used as negative control, showed no significant increase in susceptibility to HSV-1 (data not shown).

### Role of PLP in HSV-1 infection

**Fig 3:**
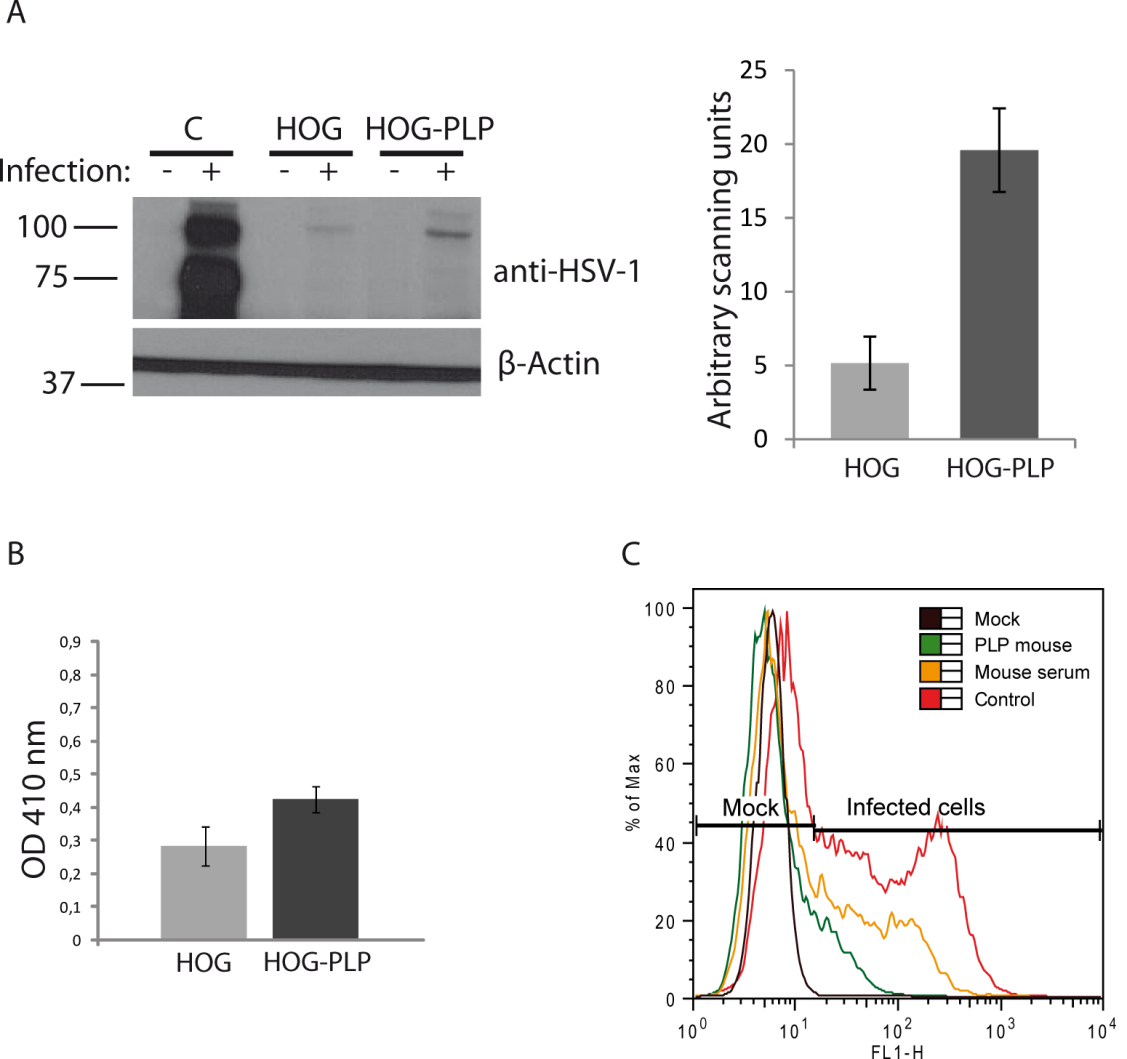
Viral entry assays. A. HOG and HOG-PLP cells were infected with R120vGF at a m.o.i. of 1. After 24 h p.i., equal number of cells were subjected to SDS–PAGE and analyzed by immunoblotting with a rabbit polyclonal anti-HSV-1 antibody to detect immediate early proteins. In HOG-PLP cells, an increase in viral signal was observed. A positive control of HOG cells infected with HSV-1 was also included. The histogram corresponds to the quantification of the immunoblot signals expressed in arbitrary scanning units. B. Confluent monolayers of cells plated in 96-well tissue culture dishes were infected with a recombinant HSV-1 (KOS) gL86 at a m.o.i. of 10. After 6 h p.i., the β-galactosidase activity at 410 nm was analyzed in a microplate reader. Optical density (OD) was increased in HOG-PLP cells compared to HOG cells. C. To perform an antibody blocking assay, HOG cells blocked with an anti-PLP antibody were infected at a m.o.i. of 1 with K26GFP and processed for flow cytometry, analyzing fluorescence of GFP. Percentage (%) of max designates the number of cells relative to the maximum fraction. For each fluorescence intensity within positive cells, the percentage of cells incubated with blocking mouse anti-PLP antibody is considerably lower than control cells incubated without blocking antibodies (red plot) or cells incubated with a control mouse serum (yellow plot). Data are representative of 3 independent experiments.

In order to investigate whether the above showed increase in viral yield was due to an increase in the viral entry, we performed an entry assay by infecting HOG and HOG-PLP cells with the recombinant R120vGF virus [29], which is unable to replicate in ICP4-defficient cells. Using the R120vGF virus, we were able to assess viral entry by detection of immediate early proteins except ICP4. HOG and HOG-PLP cells were infected with R120vGF at a m.o.i. of 1. After 24 h p.i., an equal number of cells were subjected to SDS–PAGE and analyzed by immunoblotting with anti-HSV-1 antibody to detect immediate early proteins ICP0, ICP22, ICP27 and ICP47. In HOG-PLP cells, a significant (p<0.01) 3 fold increase in viral signal was observed, suggesting a plausible PLP involvement in virus entry (Fig 3A). Subsequently, we performed an entry assay using the recombinant HSV-1 (KOS) gL86, which expresses β-galactosidase upon entry into cells. Confluent monolayers of HOG cells plated in 96-well tissue culture dishes were infected with HSV-1 (KOS) gL86 at a m.o.i. of 10. After 6 h p.i., we analyzed the β-galactosidase activity at 410 nm in a microplate reader, including HSV-1 resistant CHO-K1 cells as negative control. Again, results showed a significant (p<0.01) increase (1.5 times) in viral entry in HOG-PLP cells compared with HOG cells (Fig 3B).

Next, we carried out an antibody blocking assay. HOG cells plated in 24-well plates were preincubated at room temperature with twofold dilutions of a mouse monoclonal anti-PLP antibody for 90 min. Cells were then challenged with identical doses of the recombinant K26GFP virus–obtained by fusing GFP to the HSV-1 VP26 capsid protein- at 5 × 10^5^ pfu per well at 37°C. After 20 hours, the cells were rinsed twice with PBS and treated for flow cytometry. Pretreatment with the anti-PLP monoclonal antibody markedly reduced infection (7 times) of HOG cells by HSV K26GFP (Fig 3C). In contrast pretreatment with polyclonal serum antibodies only had a limited effect on infection. In addition, a goat polyclonal serum against PLP was also able to reduce infection of HOG cells (data not shown).

### Interaction of PLP and HSV-1

**Fig 4:**
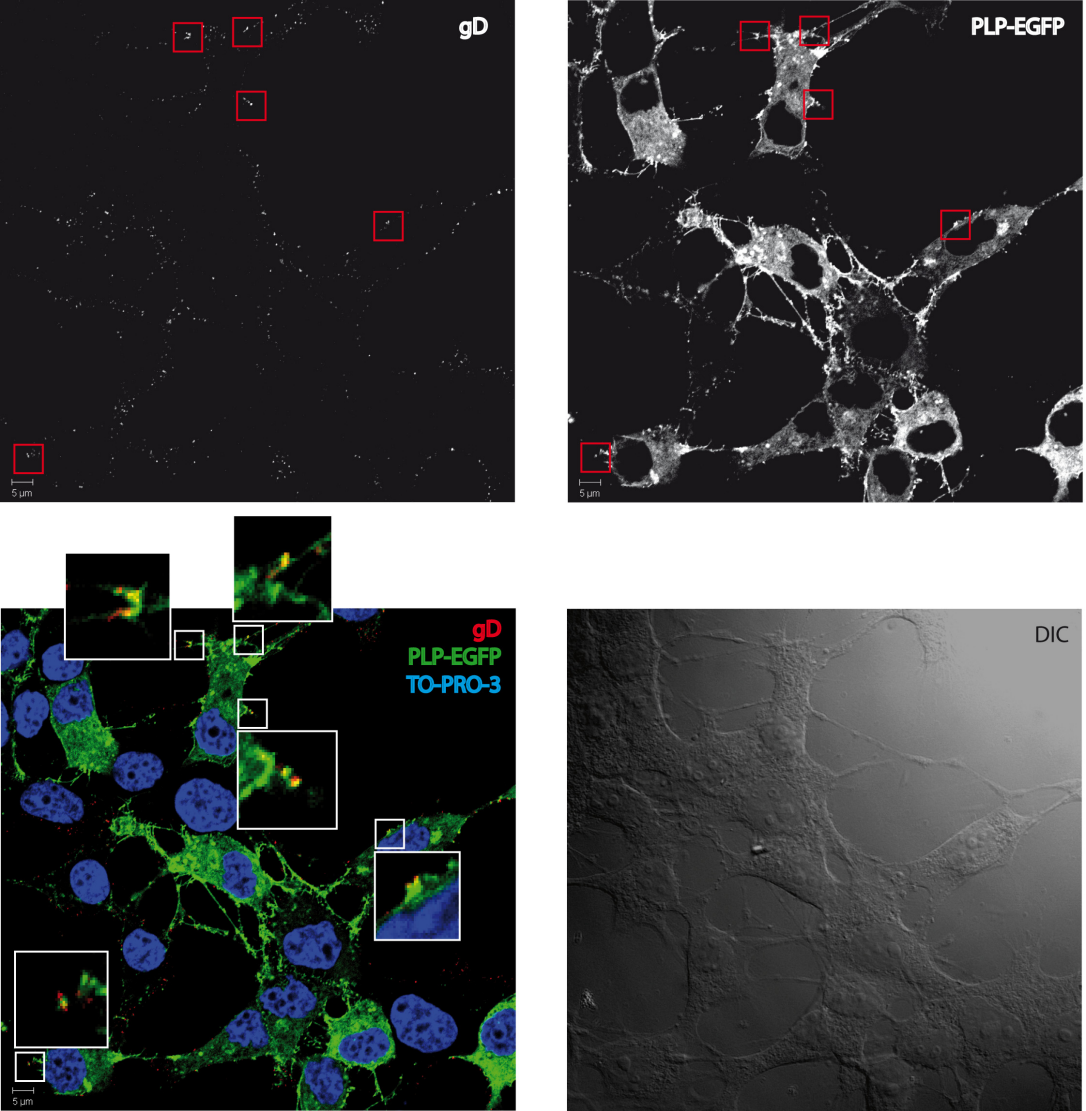
Colocalization between PLP and HSV-1. HOG cells incubated at 4°C for 1 h at a m.o.i. of 100 with HSV-1 were fixed and processed for confocal double-label indirect immunofluorescence analysis with an anti-gD LP2 antibody. Images correspond to confocal slices of 0.6 μm. Images show partial colocalization (yellow) between PLP-EGFP (green) and virions (red). (DIC: Differential Interference Contrast).

Once considered the possibility that PLP is involved in viral entry, we decided to evaluate the association between PLP and a HSV-1. To uncover such a possible relationship, we first performed an immunofluorescence assay. To that aim, we infected HOG cells with HSV-1 at a m.o.i. of 100 and subsequently incubated for 1 h at 4°C. Then, HOG-PLP cells were fixed and stained with anti-gD antibody LP2 for confocal immunofluorescence analysis. Analysis of confocal images showed partial colocalization of HSV-1 virions and PLP at patches of the cell membrane (Fig 4).

**Fig 5:**
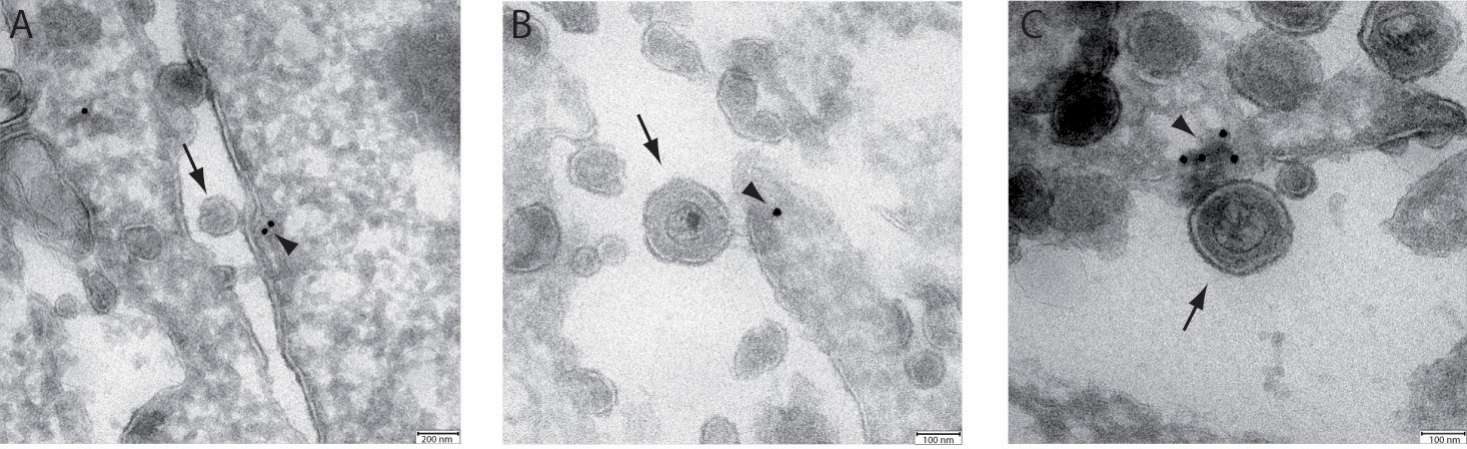
EM immunocolocalization of PLP-EGFP and HSV-1. HOG-PLP cells were mock-infected or infected with HSV-1 at a m.o.i. of 100 and incubated for 1 h at 4°C. Then, cells were fixed and processed for observation by electron microscopy. Panels A, B and C show the presence of virions (arrows) attached to the plasma membrane localized next to colloidal gold particles (arrowheads) corresponding to PLP.

The colocalization between PLP and virions was confirmed by electron microscopy. HOG-PLP cells were mock-infected or infected with HSV-1 at a m.o.i. of 100 and incubated for 1 h at 4°C. Then, cells were fixed, PLP was stained with colloidal gold-labelled antibody and cells were processed for observation as described in materials and methods. Fig 5 shows representative images of virions attached to HOG-PLP cells. The colloidal gold particles localize PLP and showed an accumulation of PLP at the site of HSV binding to the plasma membrane (Fig 5). These visual observations are consistent with our functional data in supporting a role for PLP in entry of HSV into HOG cells.

## Discussion

HSV-1 has acquired the ability to enter many different hosts and cell types by using several strategies. The most relevant factors that have allowed this ability to infect such a wide range of hosts and tissues include the number of glycoproteins involved in viral entry, the existence of multiple alternative cell receptors and the varied strategies of viral entry [35]. The two major entry pathways are fusion at a neutral pH and low-pH-dependent endocytosis [5, 35]. A third pathway, low-pH-independent gD-dependent endocytosis, has also been described [36]. In addition to the initially established cell receptors, others, such as PILRα [17], MAG [18] and non-muscle myosin heavy chain-IIA [37], have been described, and several signs suggest that more receptors for HSV-1 must exist, for instance, a gB receptor associated to lipid-rafts or gH-gL receptors.

Previous work carried out by our group [29] indicated that HSV-1 entry in oligodendrocytic HOG cells could be mediated by several possible receptors and/or by endocytic pathway and, therefore, we decided to investigate other feasible mediators of entry in our oligodendrocytic model. MAG, a myelin protein, has been proved to mediate HSV-1 entry in promyelocytes through gB interaction [18]. Nevertheless, the undetectable level of MAG expression observed in HOG cells prompted us to look for another candidate that might be implicated in HSV-1 entry. Therefore, we decided to ascertain whether PLP, the major myelin protein, could be involved in viral entry into HOG cells. As a first step, and to investigate whether overexpression of PLP was correlated with an increase in HSV-1 infection, we produced a PLP-EGFP expressing HOG cell line named HOG-PLP. Plaque assay of HOG-PLP cells infected with HSV-1 showed an increment in the number of plaques compared to HOG when cells were infected with the same viral dose. In addition, the TCID_50_/ml showed that viral yield of infected HOG-PLP cells was significantly higher compared to that of HOG non-transfected cells, an enhancement that paralleled the presence of PLP-EGFP.

To determine whether that increase in viral yield was due to an increase in the viral entry, we used R120vGF virus [29]. When the infection of HOG and HOG-PLP cells with R120vGF virus took place, we found an increase in viral signal in HOG-PLP cells, suggesting that PLP was involved in entry rather than a later step in viral replication. In addition, the fact that a mouse monoclonal antibody against PLP drastically inhibited HSV-1 entry in HOG cells suggests a direct role in the entry process. These functional data are supported by the fact that PLP and virions colocalized in confocal immunofluorescence and electron microscopy images, which showed virions attached to the plasma membrane next to PLP (visualized by colloidal gold particles). Taken together these results strongly support an involvement of PLP in the process of HSV-1 entry in oligodendrocytic cells.

The exact mechanism by which PLP enhanced HSV entry remains unclear. We observed that overexpression of PLP-GFP in the gD-receptor negative B78H1 cells did not allow entry like nectin-1 and HVEM did (data not shown) [38, 39]. This indicates that PLP does not substitute for a gD receptor such as nectin-1 or HVEM in those cells and it is unlikely that PLP acts by binding gD like the other known receptors. Nevertheless, a PLP effect on the viral attachment or the fusion process cannot be excluded. However, since we showed that nectin-1 and HVEM were functional on oligodendrocytes [29], it is possible that PLP modulates the activity of these receptors. The reported gB receptors (PILRalpha, MAG and NMHCIIA) also enhance HSV in the presence of a gD receptor. Therefore it is also possible to envisage a similar function for PLP in HSV entry into HOG cells. On the other hand, PLP might be also involved in the viral entry from the cell surface by endocytosis. Future studies will define whether PLP act similarly to those surface proteins during infection of oligodendrocytes.
